# Virtual reality education on myalgic encephalomyelitis for medical students and healthcare professionals: a pilot study

**DOI:** 10.1186/s12909-024-05990-2

**Published:** 2024-09-17

**Authors:** Tara Anderson, Grace Duffy, Dagmar Corry

**Affiliations:** School of Psychology, Queen’s University Belfast, Belfast, Northern Ireland

**Keywords:** Myalgic encephalomyelitis, Chronic fatigue syndrome, Medical education, Virtual reality, Immersive experience

## Abstract

**Introduction:**

Myalgic encephalomyelitis/ chronic fatigue syndrome (ME/CFS) is a chronic condition which may be characterised by debilitating fatigue, post-exertional malaise, unrefreshing sleep, and cognitive difficulties. ME/CFS has significant negative impact on quality of life for those living with the condition. This may be exacerbated by a lack of knowledge within healthcare regarding the condition. Previous research has found that immersive virtual reality (VR) educational experiences within healthcare education can increase knowledge and empathy.

**Methods:**

The present study employed a quasi-experimental pre-test-post-test design to investigate the impact of a short immersive VR educational experience on knowledge of ME/CFS and empathy for those living with the condition. The VR experience placed participants into a virtual scene which told real life stories of the experience of people living with ME/CFS and their families. 43 participants completed in this pilot study: 28 medical students and 15 primary care health professionals. Participants completed measures of knowledge of ME/CFS and empathy before and after engagement with the experience.

**Results:**

A statistically significant increase was found for levels of knowledge (*p* < .001, *d* = 0.74) and empathy (*p* < .001, *d* = 1.56) from pre-VR experience levels to post-VR experience levels with a medium and large effect size, respectively. Further analysis revealed no statistically significant difference between baseline levels of knowledge of ME/CFS between healthcare professionals and medical students.

**Discussion:**

The present study is the first to explore the use of this short immersive VR experience as an education tool within healthcare to increase knowledge of ME/CFS, and empathy for those living with the condition. Findings allude to the previously established lack of knowledge of ME/CFS within healthcare although promisingly the increases in knowledge and empathy found suggest that this immersive VR experience has potential to address this. Such changes found in this small-scale pilot study suggest that future research into the use of VR as an educational tool within this setting may be beneficial. Use of a control group, and larger sample size as well as investigation of retention of these changes may also enhance future research.

## Background

Myalgic Encephalomyelitis is a chronic condition which may be suspected from a presentation of debilitating fatigue, post-exertional malaise, unrefreshing sleep, and cognitive difficulties [1]. The condition was first described in the 1930s following an outbreak of similar symptoms among the staff of Los Angeles County General Hospital in 1934 [2]. Although case definitions since have differed and no one name for the condition has been agreed upon [3, 4], the present paper will use the composite term Myalgic Encephalomyelitis/ Chronic Fatigue Syndrome (ME/CFS] as is most commonly used in the literature. An estimated three million people live with ME/CFS in Europe [5]. However, prevalence estimates are also challenging as there is currently no diagnostic test nor effective treatment [6]. Recent research aims to better understand the development of ME/CFS, for example ‘DecodeME’, a large scale genome-wide association study [6]. Other research has suggested the condition is associated with viral infections [7–9].

ME/CFS has a significant impact on quality of life, with 48% of those living with ME/CFS reporting an inability to engage in any productive activity [8]. Those living with the condition scored significantly lower on health-related quality of life than the population mean and the lowest of 20 conditions, including multiple sclerosis and stroke, with reports of fewer relationships and higher unemployment [10]. Additionally, people living with ME/CFS have commonly reported experiences of minimisation of their condition by health care professionals [11]. This may be heightened by General Practitioners (GPs) lack of knowledge, and in some cases, an unwillingness to recognise ME/CFS as a genuine illness [12, 13]. For example, across a range of countries many doctors and medical students have reported that they are unsure that ME/CFS is real, while patients have reported suspicion of their condition by healthcare professionals [3, 12, 14–16].

Issues with diagnosis have been suggested to fuel the stigmatisation of the condition, with stereotypes and potential maltreatment of patients based on a lack of knowledge and awareness [11]. Hospital doctors reported a lack of formal teaching on ME/CFS, and knew little about the clinical manifestations of the condition, the appropriate management, and its impact on daily living [16]. Further, in a survey of 811 UK GPs less than half of respondents correctly identified all three key clinical features of ME/CFS [3]. GP’s and hospital doctors have also reported a lack of confidence in diagnosing and managing ME/CFS patients [3, 16, 17] .

It is evident that health care professionals show a lack of knowledge in regard to ME/CFS and research has shown minimal, if any, training on the condition. For example, an analysis of 119 medical textbooks found information on the condition on only 0.09% of pages, indicating that ME/CFS was vastly underrepresented compared to other conditions [18]. Further, only 13 of 22 UK medical schools respondents taught about ME/CFS, although no information was provided on what they taught [19]. Encouragingly, medical schools and hospital doctors are aware further training is required, and have expressed a willingness to engage with such [16, 19].

A European wide study of 23 experts in the field identified serious concerns among academics and medical experts regarding the lack of knowledge and understanding of ME/CFS among primary care physicians [20]. These experts expressed unanimous support for increased teaching on ME/CFS within undergraduate courses, postgraduate training, and specifically within primary care. Little evidence exists for the incorporation of such education however, one case study found, increases in medical students’ empathy for those living with ME/CFS and their ability to better diagnose and manage the condition were found following engagement with a learning module [21].

Empathy is an important trait of health care professionals, with physician empathy linked to increased patient satisfaction [22–24]. Virtual reality [VR] may help to promote empathy and has shown promise as an educational tool within healthcare. For example, students from various health care courses reported positive learning and increased empathy following an immersive VR experience of a patient with vision and hearing loss [25]. Nursing and midwifery students also reported increased engagement and motivation to learn following an immersive VR experience of a baby’s life in the womb [26]. Students have shown satisfaction with VR learning experiences, rating such experiences as highly valuable and requesting their inclusion in curricula [27]. Further, gains in understanding of disease processes, patients’ experience, the challenges faced by family members’, and empathetic discourse have been found following VR educational experiences [27]. The immersive element of VR education may facilitate constructivist learning experiences [28]. Constructivism emerged from Piaget’s developmental perspective and emphasises the importance of learners developing knowledge through experience rather than passively absorbing information [29]. This is in line with the effectiveness of interventions such as VR, specifically within medical education, for increasing knowledge, enthusiasm, and enjoyment [30].

Given the promise of VR as an educational tool coupled with the discussed lack of existing knowledge and stigmatisation regarding ME/CFS, the condition may be amenable to an educational intervention such as VR. the present study aims to evaluate the effectiveness of an educational immersive VR experience in increasing knowledge of ME/CFS and empathy for those living with the condition. As primary care health professionals are likely the first point of contact for someone with symptoms of ME/CFS, and medical students are likely to encounter people living with ME/CFS in their future career, this evaluation will be conducted these groups. The objectives of this study were as follows:

1. To determine if engagement with the immersive VR experience improved medical students and primary care health professionals’ levels of knowledge of ME/CFS.
2. To determine if engagement with the immersive VR experience improved participants’ levels of empathy for people living with ME/CFS.

## Methods

### Design and setting

This pilot study employed a quasi-experimental pre-test/post-test design to compare participant knowledge and empathy before and after engagement with the immersive VR experience. It was conducted by undergraduate student researchers under academic supervision and formed the basis of their degree dissertation. Questionnaires which measured knowledge and empathy were delivered immediately prior to and following the VR experience.

### Population

Recruitment was supported by Queen’s University Belfast, School of Medicine, Dentistry and Biomedical Sciences, Ulster University, School of Medicine, the Derry GP Federation, Western Health and Social Care Trust, and Western Rural Healthcare. Recruitment and testing occurred in Northern Ireland between November 2022 and February 2023. A recruitment flyer was shared by both UU and QUB Medical schools to their student portals. This flyer contained a QR code at which potential participants could register their interest, and then were contacted by a member of the research team. Primary care health professionals were recruited through opportunistic sampling in a GP surgery local to a member of the research team. Initial contact with the GP surgery was made via a letter, a member of the research team then attended the surgery to enable those working there to participate. This also led to snowball sampling of other primary care health professionals outside of this GP surgery.

### Intervention

The immersive VR experience, ‘Discover ME’, was completed using a VR headset provided by ‘Hope 4 ME and Fibro NI’, a non-profit registered charity run by patients and volunteers. The experience was created by Deepa Mann-Kler along with the charity, who granted full permission to use this for the purposes of this research. The VR experience was created as part of the charity’s awareness and education campaign. The experience lasted just under seven minutes and placed participants into a virtual scene which told real life stories consisting of an animation accompanied by audio. The VR experience enables users to hear the experiences of people living with ME/CFS and their family members while looking around a virtual scene portraying animations of the individual’s story. The stories presented incorporate facts about the condition, for example post-exertional malaise as the defining feature of the disease.

#### Survey instrument

The pre- and post-measures were designed and administered using Qualtrics. Two separate questionnaires were administered: one for medical students and one for primary care health professionals. The medical student pre-questionnaire recorded gender and year of study. Primary care health professionals were asked to indicate gender, role (e.g., GP, nurse, etc.) and their length of time working in primary care. Following these demographic questions, both questionnaires then included the 20-item knowledge of ME/CFS scale developed by experts in the field [16] (α = 0.71). This scale consisted of different types of questions; 13 true/false (e.g. “ME resolves within 6 months”), and seven multiple-choice questions (e.g. “ME affects more… Men OR Women”). Finally, an adapted version of six-item empathy scale developed by Hannans et al. (2021) was completed [25]. This scale had previously been validated by content experts [25] and was adapted to reflect a patient living with ME/CFS rather than the originally included vision impairment (α = 0.44). Each item, (e.g. “I understand the perspective of a patient living with ME”) consisted of a seven-point Likert response scale ranging from 0 - strongly disagree, to 6 - strongly agree. Both scales were adapted for healthcare professionals to ensure they reflected their current career rather than their future careers.

### Data collection

Participants attended a testing session where one researcher present. Participants were welcomed and assured that if, at any time, they became uncomfortable or did not wish to continue, they could remove the headset and inform the researcher.

Participants first completed the pre-questionnaire on Qualtrics which was accessed via a QR code presented by the experimenter and completed on the participants own device (e.g., mobile phone). A participant information sheet outlining details of the study and an informed consent form were embedded within this pre-questionnaire and participants could not progress to the questionnaire until informed consent had been obtained.

Once pre-measures had been completed a holding screen was displayed which informed the participant to let the researcher know they were ready for the VR experience. The researcher then assisted the participant to put on the VR headset and ensured they could see the title screen and hear the music. Once ready, the researcher pressed ‘play’ to begin the immersive experience. The experience did not require any movement or interaction from participants, but they could look around the virtual scene by moving their head. Therefore, participants completed the VR experience sitting down, with a member of the research team present at all times to assist the participant if they wished to take the headset off or had any difficulty with participation.

When the experience ended, participants removed the headset and returned to the Qualtrics page to complete the post-questionnaire. Finally, participants were debriefed and thanked. The entire session took 15–20 min.

### Ethics

Queen’s University Belfast, Faculty of Engineering and Physical Sciences Research Ethics Committee granted ethical approval for this study on October 28th 2022 (Ref: EPS 22_349) after considering benefits and risks and ensuring participants autonomy would be respected. All participants provided informed consent via an online form. All methods were performed in accordance with the Declaration of Helsinki [31].

### Data analysis

All analyses were conducted in SPSS version 28. Knowledge scale data were coded as zero for incorrect answers and one for correct answers; total knowledge scores represented the number of correct answers provided. Empathy scale data was coded based on the Likert response scale ranging from zero (strongly disagree) to six (strongly agree). A number of items were reverse coded to ensure higher scores reflected either more accurate knowledge or higher empathy levels.

Descriptive statistics were performed to observe demographic details of the participant sample. Two paired t-tests were then conducted to examine the change from pre-test to post-test for both the knowledge and empathy scales. Cohen’s d was calculated as a measure of effect size by dividing the difference between the pre- and post-test means by the pooled standard deviation.

Additionally, an independent samples t-test was conducted to examine any difference between medical students and primary care health professionals’ level of baseline knowledge. Due to three t-test analyses in total, a Bonferroni correction was applied to the alpha value when determining the statistical significance of the results of the analyses to reduce the risk of false positives associated with multiple comparisons [32]. Alpha (0.05) was divided by the total number of comparisons (3) to give a value of α = 0.017. Results were therefore only considered to be statistically significant if their associated p-value was 0.017 or below.

## Results

In total, 43 participants (Table 1) were recruited to evaluate the impact of the immersive VR experience on knowledge of ME/CFS and empathy for those living with ME/CFS as assessed by pre- and post-questionnaires. 28 participants were medical students while 15 were primary care health professionals (three GPs, 11 first contact physiotherapists, and one advance practice paramedic). Most participants were female (67.4%), and medical students represented the majority of the sample (65.1%). Medical students’ level of study ranged from first to fifth year with the majority in their third year of study (25.6%). Most health professionals had worked in primary care for less than five years (25.6%). Table 1 provides participant demographics.

**Table 1:** Participant descriptive statistics

Descriptive Statistics		*N*	%
Gender	Male	14	32.6
	Female	29	67.4
Role	Medical Student	28	65.1
	General Practitioner	3	7.0
	First Contact Physiotherapist	11	25.6
	Advanced Practice Paramedic	1	2.3
Level of Experience	First year medical student	5	11.6
	Second year medical student	6	14.0
	Third year medical student	11	25.6
	Fourth year medical student	1	2.3
	Working in primary care for less than 5 years	11	25.6
	Working in primary care for 5–10 years	1	2.3
	Working in primary care for more than 10 years	3	7.0

### Pre-test to post-test changes in knowledge and empathy

#### Knowledge

Due to non-normally distributed data, the non-parametric Wilcoxon signed-rank test was used to determine whether there was a statistically significant difference between pre- and post-test levels of knowledge of ME/CFS. Descriptive statistics (Table 2) show that post-test scores were higher than pre-test scores. Participants showed increased scores on post-test measures (*Mdn* = 30) compared to their mean pre-test score (*Mdn* = 26), a statistically significant increase of *Mdn* = 3, *Z* = 4.86, *p* < .001, with medium effect size *d* = 0.74.

**Table 2:** Descriptive statistics for pre-test and post-test total knowledge scores

	*N*	Range	Minimum	Maximum	Mean	Std. Deviation
Knowledge pre-test	43	18	17	35	25.93	4.35
Knowledge post-test	43	21	19	37	29.53	4.30
Valid N (listwise]	43					

#### Empathy

A paired-sample t-test was used to determine whether there was a statistically significant difference between pre- and post-test levels of empathy for those living with ME/CFS. Descriptive statistics (Table 3) show that post-test scores were higher than pre-test scores. Participants showed increased scores on post-test measures (*M* = 30.02, *SD* = 2.89) compared to their mean pre-test score (*M* = 24.37, *SD* = 3.80), a statistically significant mean increase of 5.65, 95% CI [6.79, 4.51], *t(*42) = 10.01, *p* < .001, with large effect size, *d* = 1.56.

**Table 3:** Descriptive statistics for pre-test and post-test total empathy scores

	*N*	Range	Minimum	Maximum	Mean	Std. Deviation
Empathy pre-test	43	14	16	30	24.37	3.80
Empathy post-test	43	14	22	36	30.02	2.89
Valid N (listwise)	43					

#### Medical student baseline knowledge compared to primary care health professionals

Post hoc analysis found no statistically significant difference on pre-questionnaire knowledge levels between the two groups of participants: medical students and primary care health professionals. Table 4 presents descriptive statistics for baseline knowledge levels.

**Table 4:** Descriptive statistics for pre-test knowledge scores between groups

	*N*	Range	Minimum	Maximum	Mean	Std. Deviation
Medical student pre-knowledge score	28	15	17	32	25.61	4.10
Primary care health professional pre-knowledge score	15	15	20	35	26.53	4.87

#### Summary of results

**Fig. 1:**
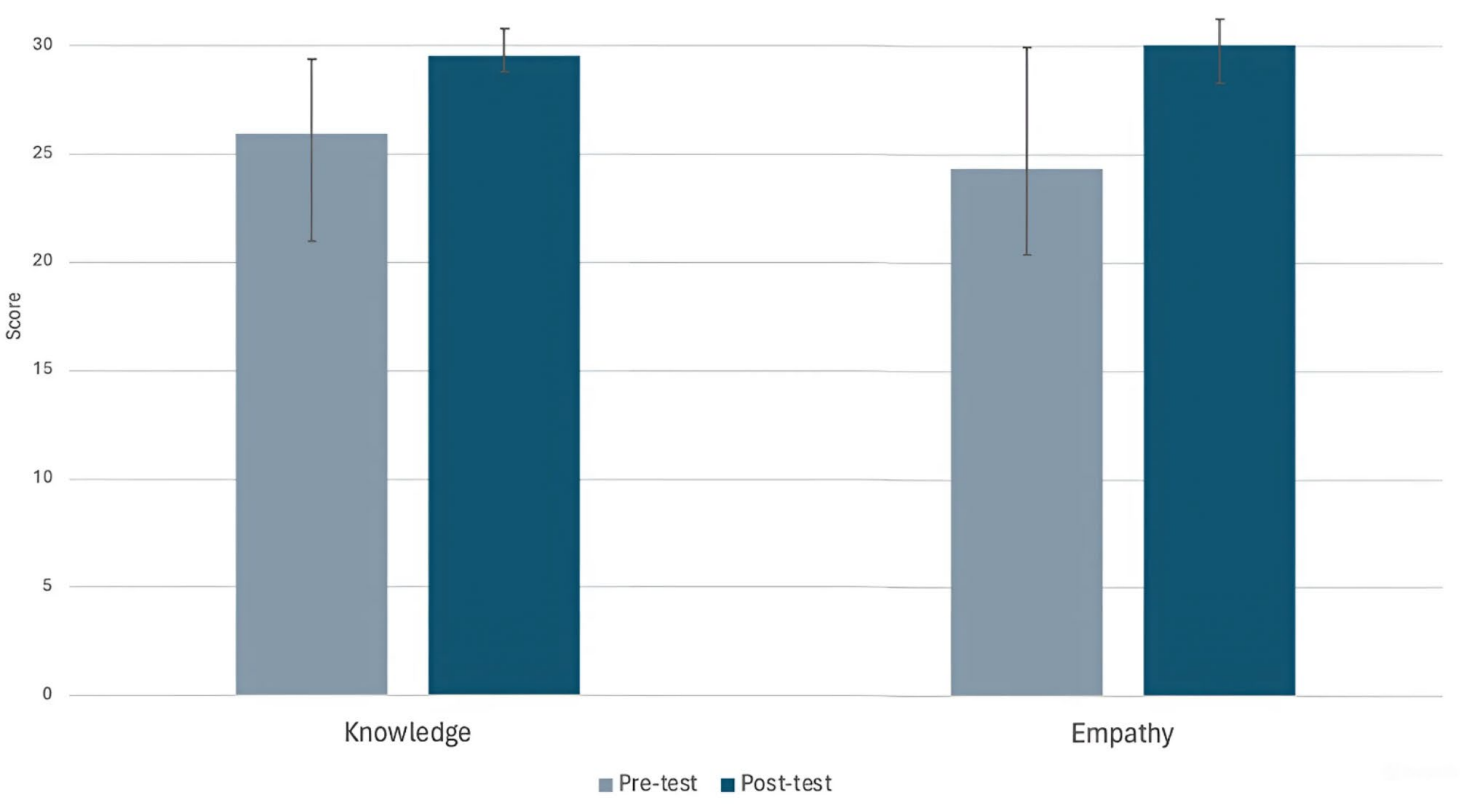
Increases in knowledge and empathy from pre- to post-test levels

To summarise, 43 participants (28 medical students and 15 primary care health professionals) were recruited to evaluate the impact of an immersive VR experience on knowledge and empathy regarding ME/CFS. Pre- and post-test analysis revealed statistically significant (*p* < .001) median increases in both knowledge of ME/CFS (with medium effect size) and empathy for those living with the condition (with large effect size) following engagement with the VR experience. These results are presented in graphical form in Fig. 1. No group differences were found on pre-test levels of knowledge.

## Discussion

ME/CFS is a debilitating chronic condition [1] of which healthcare professionals’ knowledge is lacking [3, 11, 13, 16]. GP’s and hospital doctors have, however, acknowledged their lack of confidence in the diagnosis and management of ME/CFS and expressed a willingness to engage with education initiatives [16, 17, 19]. To the best of the authors’ knowledge this is first study to investigate the utility of an immersive VR experience as an educational intervention within this context. Following engagement with the experience, participants showed both increased knowledge of ME/CFS and improved empathy for those living with the condition. These improvements highlight the potential of this experience as an educational tool for medical students and healthcare professionals.

The portrayal of individual experience of ME/CFS has been suggested to facilitate positive attitudes toward ME/CFS [3]. VR represents an innovative method to facilitate this. Existing evidence has suggested that immersive VR experiences can increase healthcare student knowledge in other contexts such as disease processes, and the experience of patients and family members [27, 30]. Taking these factors into account, the present study extends this previous literature to the context of ME/CFS as participants showed increased levels of knowledge of ME/CFS following the VR experience. Given the discussed lack of knowledge of the condition [3, 16, 17], this finding is promising as this VR experience may have potential as an educational tool for current and future health professionals.

Participants also showed increased empathy following engagement with the VR experience, although interpretations of this finding are limited due to a lack of internal validity within the scale used to measure empathy. However, increased empathy is in line with previous research regarding the utility of VR for increasing healthcare student empathy for patients with other conditions, such as those with vision and hearing loss [25, 33]. Health care professionals’ empathy has been linked to increased patient satisfaction [22–24]. This may be of increased importance within the context of ME/CFS due to patient reports of stigmatisation and suspicion from healthcare professionals regarding their condition [11, 14].

Taken together, these findings suggest that the immersive VR experience evaluated shows promise as an educational tool within medical education for medical students and primary care health professionals. The convenience of the experience, which lasts for less than seven minutes, may be beneficial to incorporate into the busy schedules of those working primary care. This may help to address concerns regarding the need for increased teaching on ME/CFS, specifically within primary care [20].

It is also interesting to note that no statistically significant difference was found between levels of knowledge measured prior to the VR experience (baseline knowledge) between primary care health professionals and medical students. Given that levels of knowledge may be expected to increase with experience level, it is surprising that those working in primary care did not show somewhat greater levels of knowledge than medical students. This finding alludes to the lack of knowledge of ME/CFS even in experienced practitioners [13, 17].

### Strengths and limitations

Both knowledge and empathy were found to increase following engagement with this immersive VR experience. This is a promising result of a short experience providing information, via real life patient stories, on ME/CFS which suggests such an educational tool may be beneficial in medical education regarding not only ME/CFS but other chronic conditions of which knowledge may currently be lacking.

Empathy was measured using an adapted version of a questionnaire developed by experts in the field of VR education [25] which was found to have a lack of internal validity as assessed by Cronbach’s alpha. This may have been a result of adapting the questionnaire to represent empathy for those living with ME/CFS. Although this questionnaire was designed specifically for pre- and post-VR experiences, future research may benefit from use of a more validated measure of empathy. Additionally, self-report measures were used which may be influenced by social desirability bias and interpretation, which has been shown particularly in relation to empathy self-reports [34, 35].

This was a small-scale pilot study, and although results are promising, the study is limited by low statistical power. This low sample size limits the generalisability of the results, however the inclusion of both medical students and health professionals working in primary care may suggest the intervention’s potential within both academic and professional medical settings. Additionally, the generalisability of the results to other healthcare professions and student groups is limited by the homogeneity of the sample. Therefore, evaluation of the experience with an increased sample size, and inclusion of more varied healthcare experiences and roles, within a randomised control trial (RCT) would facilitate more in-depth conclusions.

### Future directions

Although the increases in knowledge and empathy after a short experience are promising, it is not possible to draw conclusions on retention of this knowledge as post-test data was obtained directly after engagement with the experience. An additional questionnaire time-point sometime after the experience may help to shed light on any maintained impact of the resource on medical students’ or health care professionals’ future practice. In addition, future research may consider whether increased knowledge and/or empathy, as a result of such intervention, can positively impact patient outcomes.

The present study solely evaluated the VR experience as a tool for increasing knowledge and empathy. Although this is a convenient educational tool, it may be helpful to incorporate the experience into a more in-depth training session on ME/CFS which could lead to enhanced knowledge of the condition. It may also be helpful to compare the impact of the immersive VR experience with other interventions. Although the pre- and post-test design of the present study allowed evaluation to occur in a convenient and cost-effective manner, this design does not have a comparison or control group. Future research may benefit from comparing the results of a VR group to other interventions such as an information session, or a standalone video. Although these may be more convenient to incorporate within medical education, some of the benefits of an immersive experience may be lost. Additionally, a control group may enable conclusions regarding the causal effect of the VR experience on knowledge and/or empathy as other confounding variables could be accounted for.

## Conclusion

The use of an immersive VR experience has demonstrated increases in both knowledge of ME/CFS and empathy for those living with the condition in a participant sample of medical students and primary care health professionals in this small-scale pilot study. This represents an innovative and convenient method which may help to address gaps within medical education and improve patient experience. However, future research is needed to test the intervention with a larger population and within an RCT to provide more reliable evidence regarding effectiveness.

## Data Availability

The datasets used and/ analysed during the current study is available here: DOI: 10.17034/7b145be7-6412-4382-b93f-36fffb86e497.

## Abbreviations

ME/CFS: Myalgic encephalomyelitis/ chronic fatigue syndrome
NICE: National Institute for Healthcare and Care Excellence
GP: General practitioner
UK: United Kingdom
VR: Virtual reality
QUB: Queen’s University Belfast
QR: Quick Response
Ref: Reference
SPSS: Statistical Package for the Social Sciences
*Mdn*: Median
*M*: Mean
*SD*: Standard deviation
RCT: Randomised control trial

## References

[1] National Institute for Health and Care Excellence (NICE). Myalgic encephalomyelitis (or encephalopathy)/chronic fatigue syndrome: Diagnosis and management [Internet]. 2021 Oct. https://www.nice.org.uk/guidance/ng206 35438861

[2] Jason LA, Damrongvachiraphan D, Hunnell J, Bartgis L, Brown A, Evans M, Brown M. Myalgic encephalomyelitis case definitions. Auton Control Physiological State Function. 2012;1:1–14. 10.4303/acpsf/K110601.

[3] Bowen J, Pheby D, Charlett A, McNulty C. Chronic fatigue syndrome: A survey of GPs’ attitudes and knowledge. Fam Pract. 2005;22(4):389–93. 10.1093/fampra/cmi019. 15805128 10.1093/fampra/cmi019

[4] Lim EJ, Son CG. Review of case definitions for myalgic encephalomyelitis/chronic fatigue syndrome (ME/CFS). J Translational Med. 2020;18(1):289. 10.1186/s12967-020-02455-0. 10.1186/s12967-020-02455-0 PMC7391812 32727489

[5] Nacul L, Authier FJ, Scheibenbogen C, Lorusso L, Helland IB, Martin JA, Sirbu CA, Mengshoel AM, Polo O, Behrends U, Nielsen H. European network on myalgic encephalomyelitis/chronic fatigue syndrome (EUROMENE): Expert consensus on the diagnosis, service provision, and care of people with ME/CFS in Europe. Medicina. 2021;57(5):510. 10.3390/medicina57050510. 34069603 10.3390/medicina57050510 PMC8161074

[6] Devereux-Cooke A, Leary S, McGrath SJ, Northwood E, Redshaw A, Shepherd C, Stacey P, Tripp C, Wilson J, Mar M, Booyer D. DecodeME: Community recruitment for a large genetics study of myalgic encephalomyelitis / chronic fatigue syndrome. BMC Neurol. 2022;22(1):269. 10.1186/s12883-022-02763-6. 35854226 10.1186/s12883-022-02763-6 PMC9294749

[7] Bansal AS, Kraneveld AD, Oltra E, Carding S, What Causes MECFS. The role of the dysfunctional immune system and viral infections. J Immunol Allergy. 2022;3:1–4. 10.37191/Mapsci-2582-6549-3(2)-033.

[8] Chu L, Valencia IJ, Garvert DW, Montoya JG. Onset patterns and course of myalgic encephalomyelitis/chronic fatigue syndrome. Front Pead. 2019;7:12. 10.3389/fped.2019.00012. 10.3389/fped.2019.00012 PMC6370741 30805319

[9] Lacerda EM, Geraghty K, Kingdon CC, Palla L, Nacul L. A logistic regression analysis of risk factors in ME/CFS pathogenesis. BMC Neurol. 2019;19(275). 10.1186/s12883-019-1468-2. 10.1186/s12883-019-1468-2 PMC6839177 31699051

[10] Falk Hvidberg M, Brinth LS, Olesen AV, Petersen KD, Ehlers L. The health-related quality of life for patients with myalgic encephalomyelitis / chronic fatigue syndrome (ME/CFS). PLoS ONE. 2015;10(7):e0132421. 10.1371/journal.pone.0132421. 26147503 10.1371/journal.pone.0132421 PMC4492975

[11] Anderson VR, Jason LA, Hlavaty LE, Porter N, Cudia J. A review and meta-synthesis of qualitative studies on myalgic encephalomyelitis/chronic fatigue syndrome. Patient Educ Counselling. 2012;86(2):147–55. 10.1016/j.pec.2011.04.016. 10.1016/j.pec.2011.04.016 PMC3229648 21571484

[12] Nacul LC, Lacerda EM, Pheby D, Campion P, Molokhia M, Fayyaz S, Leite JC, Poland F, Howe A, Drachler ML. Prevalence of myalgic encephalomyelitis/chronic fatigue syndrome (ME/CFS) in three regions of England: A repeated cross-sectional study in primary care. BMC Med. 2011;9:1–2. 10.1186/1741-7015-9-91. 21794183 10.1186/1741-7015-9-91 PMC3170215

[13] Thomas MA, Smith AP. Primary healthcare provision and chronic fatigue syndrome: A survey of patients’ and general practitioners’ beliefs. BMC Fam Pract. 2005;6:1–6. 10.1186/1471-2296-6-49. 16351714 10.1186/1471-2296-6-49 PMC1325235

[14] Blease C, Carel H, Geraghty K. Epistemic injustice in healthcare encounters: Evidence from chronic fatigue syndrome. J Med Ethics. 2017;43(8):549–57. 10.1136/medethics-2016-103691. 27920164 10.1136/medethics-2016-103691

[15] Pheby DF, Araja D, Berkis U, Brenna E, Cullinan J, de Korwin JD, Gitto L, Hughes DA, Hunter RM, Trepel D, Wand-Steverding X. A literature review of GP knowledge and understanding of ME/CFS: A report from the socioeconomic working group of the European network on ME/CFS (EUROMENE). Medicina. 2020;57(1):7. 10.3390/medicina57010007. 33374291 10.3390/medicina57010007 PMC7823627

[16] Hng K, Geraghty K, Pheby D. An audit of UK hospital doctors’ knowledge and experience of myalgic encephalomyelitis. Medicina. 2021;57(9):885. 10.3390/medicina57090885. 34577808 10.3390/medicina57090885 PMC8464998

[17] Chew-Graham C, Dowrick C, Wearden A, Richardson V, Peters S. Making the diagnosis of chronic fatigue syndrome/myalgic encephalitis in primary care: A qualitative study. BMC Fam Pract. 2010;11(1):16. 10.1186/1471-2296-11-16. 20178588 10.1186/1471-2296-11-16 PMC2836312

[18] Jason LA, Paavola E, Porter N, Morello ML. Frequency and Content Analysis of CFS in Medical Text Books. Aust J Prim Health. 2010. 10.1071/PY09023. 10.1071/py09023 PMC3691015 21128580

[19] Muirhead N, Muirhead J, Lavery G, Marsh B. Medical school education on myalgic encephalomyelitis. Medicina. 2021;57(6):542. 10.3390/medicina57060542. 34071264 10.3390/medicina57060542 PMC8230290

[20] Cullinan J, Pheby DFH, Araja D, Berkis U, Brenna E, de Korwin JD, Gitto L, Hughes DA, Hunter RM, Trepel D, Wang-Steverding X. Perceptions of European ME/CFS experts concerning knowledge and understanding of ME/CFS among primary care physicians in Europe: A report from the European ME/CFS research network (EUROMENE). Medicina. 2021;57(3):208. 10.3390/medicina57030208. 33652747 10.3390/medicina57030208 PMC7996783

[21] Brimmer DJ, Jones JF, Boneva R, Campbell C, Lin JMS, Unger ER. Assessment of ME/CFS (myalgic Encephalomyelitis/Chronic fatigue syndrome): A case study for health care providers. MedEdPORTAL. 2016;10527. 10.15766/mep_2374-8265.10527. 10.15766/mep_2374-8265.10527 PMC6440494 30984868

[22] Derksen F, Bensing J, Lagro-Janssen A. Effectiveness of empathy in general practice: A systematic review. Br J Gen Pract. 2013;63(606):e76–84. 10.3399/bjgp13X660814. 23336477 10.3399/bjgp13X660814 PMC3529296

[23] Kim SS, Kaplowitz S, Johnston MV. The effects of physician empathy on patient satisfaction and compliance. Eval Health Prof. 2004;27(3):237–51. 10.1177/0163278704267037. 15312283 10.1177/0163278704267037

[24] Pollak KI, Alexander SC, Tulsky JA, Lyna P, Coffman CJ, Dolor RJ, Gulbrandsen P, Østbye T. Physician empathy and listening: Associations with patient satisfaction and autonomy. J Am Board Family Med. 2011;24(6):665–72. 10.3122/jabfm.2011.06.110025. 10.3122/jabfm.2011.06.110025 PMC3363295 22086809

[25] Hannans JA, Nevins CM, Jordan K. See it, hear it, feel it: Embodying a patient experience through immersive virtual reality. Inf Learn Sci. 2021;122(7/8):565–83. 10.1108/ILS-10-2020-0233.

[26] Hardie P, Darley A, Carroll L, Redmond C, Campbell A, Jarvis S. Nursing & Midwifery students’ experience of immersive virtual reality storytelling: An evaluative study. BMC Nurs. 2020;19(1):78. 10.1186/s12912-020-00471-5. 32821245 10.1186/s12912-020-00471-5 PMC7433077

[27] Elzie CA, Shaia J. Virtually walking in a patient’s shoes—the path to empathy? Med Sci Educ. 2020;30(4):1737–9. 10.1007/s40670-020-01101-0. 34457839 10.1007/s40670-020-01101-0 PMC8368499

[28] Marougkas A, Troussas C, Krouska A, Sgouropoulou C. Virtual reality in education: A review of learning theories, approaches and methodologies for the last decade. Electronics. 2023;12(13):2832. 10.3390/electronics12132832.

[29] Kaufman DM. Teaching and learning in medical education: How theory can inform practice. Understanding Medical Education: Evidence, Theory, and Practice. 2018;37–69. 10.1002/9781119373780.ch4

[30] Barteit S, Lanfermann L, Bärnighausen T, Neuhann F, Beiersmann C, Augmented. Mixed, and virtual reality-based head-mounted devices for medical education: Systematic review. JMIR Serious Games. 2021;9(3):e29080. 10.2196/29080. 34255668 10.2196/29080 PMC8299342

[31] World Medical Association. World medical association declaration of Helsinki. Ethical principles for medical research involving human subjects. Bull World Health Organ. 2001;79(4):373–4. 11357217 PMC2566407

[32] Bland JM, Altman DG. Statistics notes: Multiple significance tests: The bonferroni method. BMJ. 1995;310(6973):170–170. 10.1136/bmj.310.6973.170. 7833759 10.1136/bmj.310.6973.170 PMC2548561

[33] Buchman S, Henderson D. Interprofessional empathy and communication competency development in healthcare professions’ curriculum through immersive virtual reality experiences. J Interprofessional Educ Pract. 2019;15:127–30. 10.1016/j.xjep.2019.03.010.

[34] Colliver JA, Conlee MJ, Verhulst SJ, Dorsey JK. Reports of the decline of empathy during medical education are greatly exaggerated: A reexamination of the research. Academic Medicine. 2010;85(4):588 – 93. doi: 10.1097/ACM.0b013e3181d281dc. 10.1097/ACM.0b013e3181d281dc 20354372

[35] Sassenrath C. Let me show you how nice I am: Impression management as bias in empathic responses. Social Psychol Personality Sci. 2020;11(6):752–60. 10.1177/1948550619884566.

